# Social value of a set of proposals for the ideal approach of multiple sclerosis within the Spanish National Health System: a social return on investment study

**DOI:** 10.1186/s12913-020-4946-8

**Published:** 2020-02-04

**Authors:** Ester Moral Torres, Óscar Fernández Fernández, Pedro Carrascal Rueda, Elena Ruiz-Beato, Elvira Estella Pérez, Rita Manzanares Estrada, Teresa Gómez-García, Margarita Jiménez, Álvaro Hidalgo-Vega, María Merino

**Affiliations:** 1 Neurology Service, Moisès Broggi Hospital – General Hospital of l’Hospitalet, Barcelona, Spain; 2 Neuroimmunology & Multiple Sclerosis, Institute of Biomedical Research of Malaga, Malaga, Spain; 3 Directorate, Esclerosis Múltiple España, Madrid, Spain; 4 Roche Farma España, Madrid, Spain; 5 Roche Farma España, Madrid, Spain; 6 Roche Farma España, Madrid, Spain; 7 Health Outcomes Research Department, Weber, Madrid, Spain; 8 Pharmacoeconomics and Market Access Department, Weber, Madrid, Spain; 9 Economy and Health Research Seminar, Universidad de Castilla-La Mancha, Toledo, Spain; 10 Health Outcomes Research Department, Weber, Calle Moreto, 17, 5 Dcha, 28014 Madrid, Madrid Spain

**Keywords:** Multiple sclerosis, Disease management, Social value, Social return on investment, SROI, Spain

## Abstract

**Background:**

Multiple Sclerosis (MS) is a chronic inflammatory, demyelinating and neurodegenerative disease that in many cases produces disability, having a high impact in patients’ lives, reducing significantly their quality of life. The aim of this study was to agree on a set of proposals to improve the current management of MS within the Spanish National Health System (SNHS) and apply the Social Return on Investment (SROI) method to measure the potential social impact these proposals would create.

**Methods:**

A Multidisciplinary Working Team of nine experts, with representation from the main stakeholders regarding MS, was set up to agree on a set of proposals to improve the management of MS. A forecast SROI analysis was carried out, with a one-year timeframe. Data sources included an expert consultation, a narrative literature review and a survey to 532 MS patients. We estimated the required investment of a hypothetical implementation, as well as the potential social value that it could create. We calculated outcomes in monetary units and we measured intangible outcomes through financial proxies.

**Results:**

The proposed ideal approach revealed that there are still unmet needs related to MS that can be addressed within the SNHS. Investment would amount to 148 million € and social return to 272 million €, so each euro invested could yield almost €2 of social return.

**Conclusions:**

This study could guide health interventions, resulting in money savings for the SNHS and increases in patients’ quality of life.

## Background

Multiple sclerosis (MS) is the most disabling neurological disease in young adults, which causes significant limitations in patients’ personal, family, social, and work life [1–3].

The prevalence of MS in Spain has increased in recent decades from 53 patients per 100,000 inhabitants in 1994 [4], to 125 cases per 100,000 inhabitants in 2008 [5]. At present, the European Multiple Sclerosis Platform estimates that there are approximately 47,000 adult patients in Spain [6]. In parallel, an increase in incidence has been detected, with an annual average per 100,000 inhabitants of 5.3 cases between 1998 and 2003 [7], and 5.8 cases between 2008 and 2014 [8].

As with other chronic diseases, patients with MS have a high incidence of co-morbidities [9, 10], that can affect the illness by delaying diagnosis, accelerating disability, worsening quality of life, and increasing mortality [11–14]. Spanish patients with MS have an average of 5.0 ± 3.0 co-morbidities, the most frequent being depression (32.4%) and metabolic diseases such as dyslipidemia (31.1%), arterial hypertension (23.0%), obesity (22.5%), and diabetes mellitus (7.7%). In addition, 9% have chronic obstructive pulmonary disease, and 6.3% have asthma [15].

The disability derived from these factors contributes to the deterioration of health-related quality of life (HRQoL) of patients with MS [16, 17]. In 2017, Spanish patients, via the *EuroQol-5 Dimensions* (EQ-5D) questionnaire, reported pain and discomfort (63%), problems in carrying out daily activities (62%), anxiety / depression (55%), mobility difficulties (54%), and self-care problems (26%) [18]. Only 45% of patients with MS of working age were employed or self-employed and, among those employed, 72% felt that MS affected their productivity, mainly due to fatigue (64%), difficulty thinking (29%), moodiness (27%), mobility (25%), and pain (20%) [18].

MS also impacts the HRQoL of their informal caregivers: 20.6% have symptoms of depression, 10.6% perceive their family as dysfunctional, and 9.4% receive little social support [19]. Likewise, patient’s progressive increase in cognitive deficit causes a higher incidence of depressive symptoms in caregivers, further contributing to the deterioration of the family environment [20].

The healthcare needs of patients with MS depend on the symptoms, the degree of disability, and the existence of co-morbidities [1, 10]. Since it is a chronic disorder, care must include patients as well as their relatives and caregivers [21].

The early diagnosis of MS has been identified as one of the main needs: in Spain, the average onset age of the first symptoms is 31.4 years, while the average age at the time of diagnosis is 33.6 years [8]. Accordingly, a diagnostic delay of more than 2 years is estimated [8, 22]. Furthermore, the twenty-first Century Steering Group*,* comprising patients and healthcare professionals, detected unmet MS health needs regarding symptom management, treatment access, patient access to information, and communication between patients and health professionals [23].

The Social Return on Investment (SROI) method, developed in 1996 by the Roberts Enterprise Development Fund, aimed to account for the social value of interventions, offering a framework to measure returns that do not have a market value but possess an intrinsic value (e.g. emotional well-being of patients or satisfaction with the healthcare system) [24, 25]. The current SROI method further includes principles and processes typically used in evaluations of economic and financial return on investment [26]. The SROI method has not been applied to the management of MS previously, however, the methodology has been used in the area of neurology [27, 28], as well as other areas such as dermatology, cardiology, rheumatology, and oncology within the SNHS [29–33], and other health-related areas in other countries (nephrology [34], old age [35, 36], or maternity [37, 38], among others [39, 40]).

Thus, the objective of this study was twofold: first, to agree on a set of proposals that contribute to the ideal approach to MS in the Spanish National Health System (SNHS) and, secondly, to analyse the potential social value that would be created after its implementation.

## Methods

**Fig. 1:**
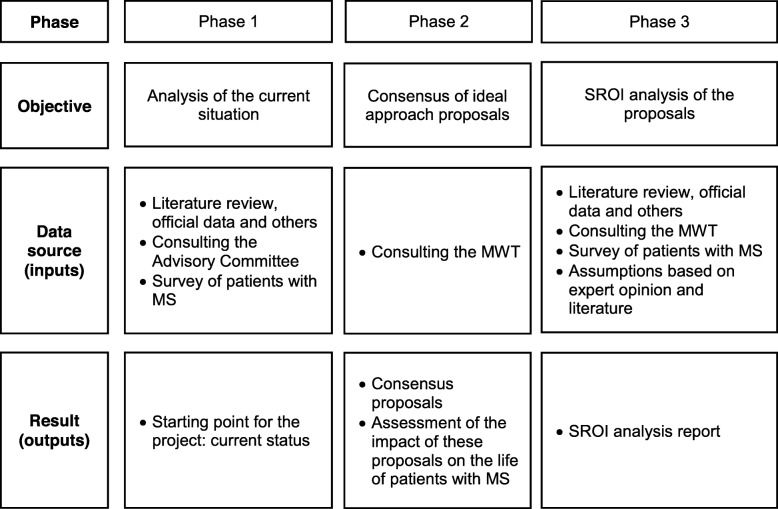
Work process of the Project. Abbreviations: MS, multiple sclerosis; MWT, Multidisciplinary Working Group; SROI, social return on investment

The project was developed according to the following phases (Fig. 1):

- Phase 1 (initial): description of the current approach to MS in the SNHS, as well as the affectation suffered by MS patients as a starting point for the study to be performed.
- Phase 2 (first objective, ideal approach): definition of proposals contributing to the ideal approach to MS in the SNHS.
- Phase 3 (second objective, social return on investment [SROI] analysis): analysis of the SROI of the hypothetical implementation of the previous proposals, based on the principles and stages proposed by the SROI guide [41].

The following data sources, which were developed by the authors for the purposes of this study, were used:

1. Narrative literature review.
  Scientific articles, official data and, to a lesser extent, grey literature (mainly news and websites regarding MS) was reviewed.
  The review helped establish the current approach to MS in the initial phase of the Project and provided information for the analysis phase.
2. Survey of patients with MS.
  A survey was conducted on 532 adult patients with MS living in Spain, through an on-line electronic questionnaire, between June and July 2017. An English translation of the questionnaire can be found in the Additional file 1.
  The results provided information about the needs and HRQoL of patients with MS in the initial phase of the Project, as well as quantitative information for the analysis phase to carry out calculations.
3. Expert consultation.
  A Multidisciplinary Working Team (MWT) of 9 experts was set up, with representation from the main stakeholders regarding MS: 2 from neurology, 1 from primary care medicine, 1 from specialist nursing, 1 from hospital pharmacy, 1 from physiotherapy, 1 from social work, 1 from association of patients, and 1 patient with MS.
  Three of the nine experts participated as an Advisory Committee in establishing the current approach to MS (starting point), and together with the rest of the experts, agreed on a set of proposals aimed at achieving the ideal approach to MS (first objective). Subsequently, the MWT assessed the impact of each proposal on each life areas of the MS patient, which helped establish the potential returns of each proposal.

In order to identify an ideal approach to the management of MS, an 8-h meeting with the MWT was held. In this meeting, three work subgroups were organised according to the individual perspective of the experts: medicine (neurology and primary care), other health professionals (specialist nursing, hospital pharmacy and physiotherapy) and patients (social work, patients association, and patient).

Each group had a predetermined time to internally discuss the most relevant proposals for the ideal approach to MS. Thereafter, proposals were shared with the rest of the groups via a spokesperson. The proposals were discussed and collected around three categories that were previously established by the Advisory Committee: diagnosis, relapsing-remitting MS, and progressive forms of MS (which encompass primarily progressive MS and secondarily progressive MS).

Next, the MWT was asked to rate the proposals individually according to the importance they considered each proposal to have for an ideal approach to MS, on a scale from 0 (“not important”) to 10 (“maximum importance”). Finally, based on the basic principle of the economy of resource scarcity, the 6 proposals with the highest average score in each area were selected.

Regarding the second objective, the forecast type SROI method was applied, with a one-year timeframe. In order to determine investment, the SNHS perspective was used, while impact was determined from a social perspective. The analysis combined both qualitative and quantitative methodologies, as dictated by the SROI guide [41].

The qualitative analysis implied understanding how the set of proposals put forward by the MWT would create social value after its hypothetical implementation, that is, the process by which each investment would generate a return, which is called the Theory of Change according to the SROI method [41].

The identification of these processes was based, first, on the opinion of the MWT that assessed the importance of each proposal in various areas of the patient’s life and, in turn, on the literature review.

The quantitative analysis focused on the process of calculating investment, return, and impact. In order to calculate the investment, the activities necessary to implement each proposal, the necessary resources, and the cost associated with those resources were first identified. Thereafter, these resources were multiplied by their unit prices. Resources, be they medical or non-medical, material or human, were quantified (in number and cost) from the literature review, official data, public prices of health services of each of the Spanish autonomous regions, and market prices. No financial value was given to the time considered for patients and their caregivers, since they are the main beneficiaries of the project, following the current SROI methodology convention [41].

Return was calculated by identifying the potential consequences of each proposal in clinical, welfare, economic, and social terms. Returns, be they tangible or intangible, positive or negative, were identified from the expert opinion of the MWT, and from the literature review, official data, public prices of health services of each of the Spanish autonomous regions and market prices. The increase or decrease in the burden of care that informal caregivers would assume was quantified using the substitution cost method, which consists of allocating the cost of hiring a professional caregiver for the time spent for informal care. Moreover, losses or gains in labour productivity were measured using the human capital method, assigning the average wage cost lost/earned as a consequence of the proposal. The intangible returns (those that do not have a market price) were quantified by assigning financial proxies such as revealed preferences (for example, the proxy of being well informed could be equivalent to the fee paid by the partners of an association of patients) or declared preferences (for example, the willingness to pay to improve their emotional state declared by patients with MS in the survey).

To adjust the total impact of the return, the deadweight (percentage of the return that would have been obtained without the proposal), the attribution (percentage of the return resulting from other activities independent from the proposal), the displacement (percentage of the return that would have displaced another return), and the drop-off (percentage of return deterioration over time)^1^were deducted. Information on adjustment factors was obtained from literature review, survey to MS patients, and expert opinion.

Prices were updated to euros from 2017 according to the corresponding Consumer Price Index [42]. Regarding missing data, some assumptions based on expert opinion and literature were made, such as the average number of extra medical visits required, the average needed time for every medical visit, or the number of neurologists to be trained in specialized MS units, among others.

All calculations were based on the prevalence reported by the MS Barometer 2015 [6], updated to the population figures of 2017 (47,084 patients with MS in Spain) and an incidence of 2701 patients according to published data [8]. We assumed the entire population of MS patients would adhere to the set of proposals. Spain is geographically divided into 17 autonomous communities with decentralized management of health services, hence some proposals here presented may already have been implemented in some regional health services, but not in others.

The SROI ratio was calculated by dividing the total estimated impact by the estimated necessary investment, and so the analysis can be summarised in one sentence: “for each euro invested, a social return of X euros would be obtained”. Any ratio greater than 1 is positive.

**Fig. 2:**
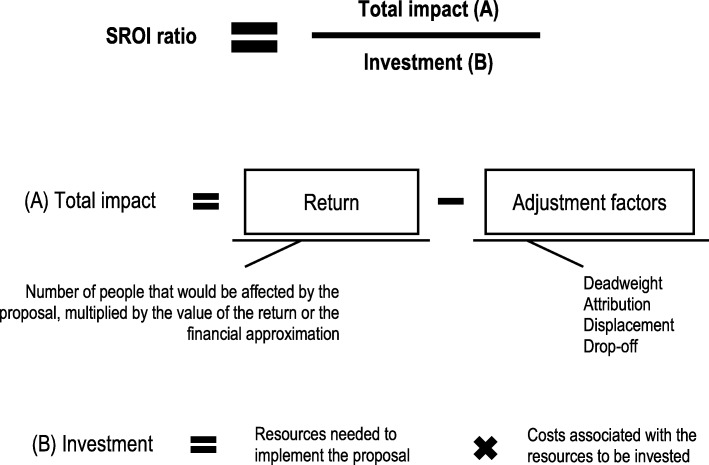
Process of calculating the investment, the return, the impact and the SROI ratio. Abbreviations: SROI, social return on investment

Figure 2 explains the process of calculating the investment, the return, the impact and the SROI ratio.

Finally, a sensitivity analysis was carried out, by varying the variables that included some type of assumption (Table 1). Thus, two alternative scenarios (best and worst) were considered as the reference case, following expert opinion.

**Table 1:** Percent variation in the assumptions considered in the Project

Assumptions included in the calculations	Reference case	Worst scenario	Best scenario
1. Percentage of disability reduction from moderate to mild in incident patients, as a consequence of the reduction in the time to diagnosis.	50%	25%	75%
2. Percentage of disability reduction from severe to moderate in the incident patients, as a consequence of the reduction in the time to diagnosis.	50%	25%	75%
3. Percentage of cross-consultations avoided in neurology after direct consultation between Primary Care and Specialised Care professionals.	50%	25%	75%
4. Percentage of reduction of informal care hours, as a result of a better follow-up of patients and the slowing down of the progression of their illness.	20%	10%	30%
5. Decrease in the percentage of patients who do not work because of MS.	20%	10%	30%
6. Percentage of untreated SPMS patients, who could be treated.	50%	75%	25%

Abbreviations: *MS* multiple sclerosis, *SPMS* Secondary Progressive MS.

Due to the type of study, no clinical research ethical committee approval was required.

## Results

### Consensus for an ideal approach to MS

A total of 18 proposals were obtained to contribute to an ideal approach to MS within the SNHS (Table 2).

**Table 2:** Proposals for the ideal approach to MS

Analysis area	Number	Proposal name
Diagnosis	1	Training in MS and its symptoms both for non-specialist MS neurology and for healthcare professionals from other areas related to MS patients.
	2	Coordination between primary care medicine and neurology, through direct contact channels.
	3	Decrease in waiting lists in the neurology speciality.
	4	Quick access to the magnetic resonance imaging test.
	5	Visit of diagnostic test results within a maximum 30 days.
	6	Early visit with neurology after diagnosis.
Relapsing-remitting MS	7	Coordination between primary care medicine and neurology, through direct contact channels.
	8	Protocol on the follow-up of patients according to the criteria of disease severity.
	9	Magnetic resonance imaging performed at least once a year.
	10	Universal access to monographic consultations and/or multidisciplinary units of MS throughout the National Health System.
	11	Access to disease modifying treatment for patients with RRMS not currently treated.
	12	Education about healthy habits for patients through hospital nursing specialised in MS.
Progressive forms of MS	13	Coordination between primary care medicine and other specialists involved in the follow-up of the disease, through direct contact routes.
	14	Care and treatment of collateral symptoms and education for their management.
	15	Access to treatment for patients with PFMS not currently being treated.
	16	Universal access to comprehensive rehabilitation.
	17	Improvement in social protection, ensuring direct contact with social work.
	18	Research on the pathogenesis of progression at a clinical and basic level (neuroprotection and remyelination). ^a^

Abbreviations: *MS* multiple sclerosis, *RRMS* relapsing-remitting MS, *PFMS* progressive forms of MS, which include both primary progressive MS and secondary progressive MS.

^a^Despite the consensus of the Multidisciplinary Working Group on the inclusion of this proposal in the ideal approach to MS in the SNHS, the impossibility of estimating neither its investment nor its potential return has led to removing it from the calculation of the social return that such an approach would entail after its hypothetical implementation

The main returns derived from the implementation of the diagnostic area proposals would be to avoid diagnostic errors, reduce the time of diagnosis, delay the evolution of the disability, improve the degree of knowledge of the patient about the disease, and reduce the emotional burden of the patient.

Regarding the area of relapsing-remitting MS, the main returns would be to avoid unnecessary visits to the neurology department, reduce relapses or outbreaks, improve treatment adherence, reduce disease progression, and improve emotional status, autonomy, quality of life, and the self-care of patients.

Finally, the proposals of the area of the progressive forms of MS would help avoid unnecessary neurology department visits, improve labour protection linked to MS, reduce outbreaks and costs per patient thanks to early treatment, and improve the quality of life, motor status, fatigue, family relationships, and the emotional state of patients.

From a global point of view, the improvement of the quality of life of the patient and the efficient use of health resources would imply that the patient did not have to lose hours of work for medical assistance and that the burden for informal caregivers would decrease. However, the proposals that involve completing more visits or medical tests would mean an increase in labour productivity losses, as well as in the care burden of their informal caregivers.

Table 3 shows the main stakeholders of each proposal, the objective sought, and the associated returns.

**Table 3:** Theory of the change in proposals for the ideal approach to MS

Proposal	Objective and Activity	Stakeholders	Expected returns according to each stakeholder^a^
1. Training in MS and its symptoms both for non-specialist MS neurology and for healthcare professionals from other areas related to MS patients.	**Objective**: To provide minimum training in EM to all health professionals who may be involved in the detection of a case of MS. **Activity**: • Design and delivery of an accredited continuous training course in each of the SNHS hospitals with a neurology department, for neurology professionals who are not specialised in MS. • Design of an accredited on-line ongoing training course, aimed at other healthcare professionals in areas related to MS.	• National Health System. • Neurology professionals not specialised in MS. • Other health professionals linked to the management of MS. • Incident patients with MS.	National Health System • Diagnostic errors would be avoided by training health professionals who treat patients with MS in the disease. Incident patients with MS • Training health professionals who care for MS patients in the disease would reduce the time to diagnosis.
2. Coordination between primary care medicine and neurology, through direct contact channels.	**Objective**: To improve coordination between PCM and neurology for an earlier diagnosis. **Activity**: Promotion of the figure of the professional consultant neurologist, so that each PCM can contact the neurology professional at the reference hospital directly, mainly by phone or through the digital medical records.	• National Health System. • Primary Care Medicine. • Neurology professionals. • Incident patients with MS.	National Health System • The waiting time for the first visit with the neurology professional would be reduced: a possible visit of the patient to Accident and Emergency department would be avoided. Incident patients with MS • It would reduce the patient’s time to diagnosis (early diagnosis) by improving communication between primary care professionals and neurologists. • The emotional state, linked to previous returns, would be improved.
3. Decrease in waiting lists in the neurology speciality.	**Objective**: To facilitate the early diagnosis of MS. **Activity:** • Modification of the appointment management tool that allows for preferential coding from PCM for suspected disease to be included in the cross-consultation for the neurology professional. • Warning, through the appointment management system, about the existence of prioritisation of suspected MS through a code.	• National Health System • Incident patients with MS	National Health System • An early MS diagnosis would delay the disability progression from mild to moderate. By reducing the referral time to the neurology professional, an early diagnosis would be reached, which would result in delaying the disability progression. • An early MS diagnosis would delay the disability progression from moderate to severe. By reducing the referral time to the neurology professional, an early diagnosis would be reached, which would result in delaying the disability progression. Incident patients with MS • Reducing the time of referral to the neurology professional would reduce the time to the diagnosis of MS, since it is one of the factors that influence the diagnosis delay.
4. Quick access to the magnetic resonance imaging test.	**Objective**: To facilitate the early diagnosis of MS. **Activity**: Extension of the magnetic resonance imaging (MRI) test schedule to weekends, for nine and a half months, of all the equipment available in SNHS hospitals with a neurology department.	• National Health System • Radiology professionals • Radiodiagnosis technicians • Incident patients with MS • Other neurological patients • Other non-neurological patients	National Health System • An early MS diagnosis would delay the disability progression from mild to moderate. • An early MS diagnosis would delay the disability progression from moderate to severe. Incident patients with MS • Reducing the waiting list for MRI would shorten the time to the diagnosis of MS. The availability of diagnostic tools is another reason for diagnosis delay. The extension of non-working days to perform the MRI test has already been carried out on a pilot basis in some hospitals, obtaining a reduction around 30% in the waiting list.
5. Visit of diagnostic test results within a maximum 30 days.	**Objective**: To facilitate the early diagnosis of MS. **Activity**: Modification in the appointment request system which allows that appointments for all the diagnostic tests can be set on a same day or a maximum of 2 days.	• National Health System • Neurology professionals • Incident patients with MS	National Health System • An early MS diagnosis would delay the disability progression from mild to moderate. • An early MS diagnosis would delay the disability progression from moderate to severe. Incident patients with MS • Reducing the waiting list for diagnostic tests would shorten the time to the diagnosis of MS. The availability of diagnostic tools is another reason for diagnosis delay.
6. Early visit with neurology after diagnosis.	**Objective**: To improve information and emotional support in the diagnosis of MS. **Activity**: Additional follow-up visit with neurology.	• Neurology professionals • Incident patients with MS • Informal carers	Incident patients with MS • The degree of patient’s understanding of the disease from the time of diagnosis would be improved. In an early visit after the diagnostic visit, patient information would improve as it would help resolve doubts. • The emotional burden of the patient at the time of diagnosis would be reduced by resolving doubts. • Labour productivity losses would occur in working patients, as a consequence of attending this visit. Informal carers • The burden of care for informal caregivers would be increased by having to accompany patients to this visit.
7. Coordination between primary care medicine and neurology, through direct contact channels.	**Objective**: To improve the quality of care for patients and avoid unnecessary displacements or erroneous referrals. **Activity**: Promotion of the figure of the professional consultant neurologist, so that each PCM can contact the neurology professional at the reference hospital directly, mainly by phone or through the digital medical records.	• National Health System • Primary Care Medicine • Neurology professionals • Patients with RRMS • Informal carers	National Health System • Unnecessary visits to neurology professionals would be avoided for RRMS patients. Patients with RRMS • The labour productivity of patients with RRMS who work would be improved by not having to go to unnecessary visits with the neurology professional. Informal carers • The burden of care for informal caregivers would be reduced by not having to accompany patients to unnecessary medical visits.
8. Protocol on the follow-up of patients according to the criteria of disease severity.	**Objective**: To improve the efficiency of healthcare processes by ensuring the application of monitoring and treatment protocols to patients with RRMS. **Activity**: 243 talks given by members of the CSURs and/or members from demyelinating diseases groups from each Autonomous Community, according to the established local protocols, for MS care, aimed at both neurology and PCM professionals.	• National Health System • Staff of the CSUR in MS and/or members from demyelinating diseases groups from each Autonomous Community • Neurology professionals specialised in MS • Primary Care Medicine • Patients with RRMS	National Health System • The number of relapses would be reduced in patients not currently treated according to the protocols. Patients with RRMS • Relapses would be avoided as a result of the appropriate approach.
9. Magnetic resonance imaging performed at least once a year.	**Objective**: To improve hospital availability of the MRI test, which allows to annually review brain lesions in patients with RRMS and assess disease activity (prognosis and progression) and/or suboptimal responses to treatments. **Activity**: Performing an imaging test, brain MRI, annually on all those patients with RRMS who are not currently being tested.	• National Health System • Neurology professionals specialised in MS • Radiology professionals • Patients with RRMS • Informal carers	National Health System • Flare-ups would be prevented in patients not undergoing an annual MRI. The follow-up of the patients and the adequacy of the treatment would be improved. Patients with RRMS • The emotional state of patients with this affected dimension would be improved, linked to the previous return. • There would be losses of labour productivity in working patients, for undergoing the MRI test. Informal carers • The care burden of informal caregivers would be increased by accompanying patients to the MRI test.
10. Universal access to monographic consultations and/or multidisciplinary units of MS throughout the National Health System.	**Objective**: To care for patients with RRMS in a more efficient way and with better quality. **Activity**: Creation of two types of resources: 1. Monographic consultations in hospitals with a neurology department that has less than 200 beds. Patients with mild RRMS would benefit from them. In this context, two visits per year to specialist MS neurology are considered. 2. Multidisciplinary MS units in the rest of the hospitals with a neurology department, with more than 200 beds. Patients with moderate and severe RRMS would benefit from them. In this case, the following is considered for each unit: • Training a specialist neurologist in MS in unit management. • Three visits per year to neurology and nurses specialised in MS, for patients with moderate RRMS. • Six visits per year to neurology and nurses specialised in MS, for patients with severe RRMS. • If required, ten visits per year to neuropsychology and sixty to neurophysiotherapy.	• National Health System • Neurology professionals specialised in MS • Nurses specialised in MS • Other specialities: neurophysiotherapy and neuropsychology • Patients with mild RRMS • Patients with moderate-severe RRMS • Informal carers	National Health System • Treatment adherence in patients with moderate-severe RRMS would be improved, mainly due to the monitoring carried out by the hospital nurses. • All patients with RRMS who did not receive drug therapy previously because they did not attend the monographic consultations/MS units would be then adequately treated. Patients with mild RRMS • The emotional state of patients with this affected dimension would be improved when receiving a better follow-up. • There would be losses in labour productivity in working patients as a result of attending visits. Patients with moderate-severe RRMS • Autonomy and quality of life would be improved due to the comprehensive approach of the multidisciplinary units. • The emotional state of the patients with this affected dimension would be improved. • There would be losses in labour productivity in working patients as a result of attending visits. Informal carers • The care burden of informal caregivers to patients with RRMS would be reduced as disease progression can be slowed down. • The care burden of informal caregivers would increase when accompanying patients with RRMS to visits.
11. Access to disease modifying treatment for patients with RRMS not currently treated.	**Objective**: To establish early treatment for patients with RRMS. **Activity**: Treatment of patients with RRMS not currently treated with hospital DMTs, as a consequence of the adverse drug reactions or of the concomitant diseases they suffer, that prevent them from receiving certain treatments.	• National Health System • Regional health services in the autonomous regions. • Neurology professionals specialised in MS • Hospital pharmacy • Patients with untreated RRMS	National Health System • The evolution of MS disability in patients with RRMS would be slowed, since they would be treated from the beginning of the diagnosis. Patients with untreated RRMS • Flare-ups would be avoided with the early pharmacological treatment.
12. Education about healthy habits for patients through hospital nursing specialised in MS.	**Objective**: To improve the quality of life of patients through changes in life habits **Activity**: 1. Group meetings led by hospital nurses, aimed at about ten patients per meeting, for training about healthy habits. 2. Printing and sending information brochures to hospitals that lack consultations/specialist units for MS	• National Health System • Nurses specialised in MS • Patients with RRMS • Informal carers	Patients with RRMS • Self-care of patients with RRMS would be improved, and they would have a healthier life, allowing patients to pay more attention to maintaining healthier lifestyles in those cases that do not. • There would be losses in labour productivity in working patients when going to consultations with hospital nurses. Informal carers • The emotional state would be improved in informal caregivers of patients with moderate-severe RRMS who have this affected dimension. • The care burden of informal caregivers would be increased by accompanying patients with RRMS to healthy habits visits.
13. Coordination between primary care medicine and other specialists involved in the follow-up of the disease, through direct contact routes.	**Objective**: To improve the quality of care for patients and avoid unnecessary displacements or erroneous referrals. **Activity**: Promotion of the figure of the consultant specialist, in such a way that each PCM can contact the corresponding professional at its reference hospital, directly, mainly by telephone or through the digital medical record.	• National Health System • Primary Care Medicine • Health specialists involved in monitoring the disease • Patients with PFMS • Informal carers	National Health System • Unnecessary visits to neurology professionals by PFMS patients would be avoided. Patients with PFMS • Labour productivity would be improved in those working patients by not having to complete unnecessary visits. Informal carers • The care burden for the caregivers would be reduced, since they do not have to accompany the patients to unnecessary visits.
14. Care and treatment of collateral symptoms and education for their management.	**Objective**: To control the collateral symptoms suffered by patients with PFMS. **Activity**: For each patient with PRMS, completing four visits per year to specialist nurses specifically aimed at this objective.	• Nurses specialised in MS • Patients with PFMS • Informal carers	Patients with PFMS • The loss of employment linked to MS in working-age patients would be reduced. The main reasons MS patients attribute to job loss are related to the ineffective management of MS symptoms in the workplace, rather than factors directly related to the workplace. • Quality of life would be improved through the improvement of urinary symptoms. We have highlighted this co-morbidity since it is associated with a great loss of quality of life. • The emotional state of patients with PFMS, linked to previous returns, would be improved. • There would be losses in labour productivity in working patients, as a consequence of attending these visits. Informal carers • The burden of care for patients with PFMS would be reduced by slowing down disease progression. • The burden of caring for caregivers would be increased, linked to them accompanying patients to the visits.
15. Access to treatment for patients with PFMS not currently being treated.	**Objective**: To provide early treatment of patients with PFMS. **Activity**: Treatment of patients with PFMS who do not currently receive hospital DMTs. This proposal only includes the treatment of patients with PFMS, since patients with PPMS do not currently have any drug with an indication for their typology.^b^	• National Health System • Regional health services in the autonomous regions. • Neurology professionals • Hospital pharmacy • Patients with PFMS	National Health System, Regional Health Services of the Autonomous Regions and the Hospital Pharmacy • The total costs would be reduced when treating patients with SPMS, that is currently untreated. If a treatment allows no progression in the disability, it is possible to calculate the difference between the cost of treating a moderate patient versus treating a mild patient. Patients with PFMS • Flare-ups would be avoided with the early pharmacological treatment.
16. Universal access to comprehensive rehabilitation.	**Objective**: To improve physical, cognitive, psychic symptoms ... that ultimately improves the disability and quality of life of patients. **Activity** access of all patients with FPMS to the following resources, if required: • 1 annual visit to a neuropsychologist • 10 annual visits to a psychologist • 60 physiotherapy sessions per year • 12 annual sessions of occupational therapy • 1 annual visit to a speech therapist • 1 annual visit to a social worker (broken down in Proposal 17)	• Neuropsychology, psychology, physiotherapy, occupational therapy, speech therapy and social work professionals • Patients with PFMS • Informal carers	Patients with PFMS • The emotional state of the patients would be improved by reducing anxiety, a consequence of visits to the neuropsychology and psychology departments. • The motor status of the patients would be improved, as a result of visits to physiotherapy. • Fatigue would be reduced in patients with PFMS. • There would be losses in labour productivity in working patients, as a consequence of attending these visits. Informal carers • The burden of patient care would be reduced as a result of improved motor status. • The burden of patient care would be increased, linked to accompanying patients to the visits.
17. Improvement in social protection, ensuring direct contact with social work.	**Objective**: To improve the social protection of patients, through the detection, assessment and diagnosis of the needs linked to MS and the disability status. In addition, to facilitate the link with MS societies as advocators for the social needs and QoL services provider. **Activity**: An annual visit to the social work service for all patients with PFMS.	• Social work professionals • Patients with PFMS • Informal carers • MS societies	Patients with PFMS • If they were recognised as having at least 33% disability, unemployed PFMS patients would improve their work productivity as a result of MS, since they could access a reserved position and working PFMS patients would maintain their labour productivity. • The work environment would be improved, from the subjective perception of the patient. • Mobility would be improved, from the subjective perception of the patient. • Family relationships would be improved, from the subjective perspective of the patient. • There would be losses in labour productivity in working patients, as a consequence of attending these visits. Informal carers • The burden of care would be reduced, in relation to the improvement of the patient. • The burden of caring for caregivers would be increased, linked to them accompanying patients to the visits.

Abbreviations: *MS* multiple sclerosis, *PCM* medicine / primary care physician, *PC* primary care, *MRI* magnetic resonance imaging, *RRMS* relapsing-remitting MS, *CSUR* Reference Centres, Services and Units, *SPMS* secondarily progressive MS, *PPMS* primarily progressive MS, *PFMS* progressive forms of MS, which include both primary progressive MS and secondary progressive MS.

^a^ Although the stakeholders of each proposal are affected, this column includes only the returns that have been quantified in the SROI analysis, as they are the most relevant

^b^The first and for the moment the only MS treatment for PPMS is already authorised by the European Medicines Agency

### Impact of the proposals for the ideal approach to MS

The total amount of resources invested by all the stakeholders in the set of proposals for the ideal approach to MS would amount to 148.35 million euros. Most of the investment would focus on the areas of the progressive forms of MS (52.4%) and relapsing-remitting MS (43.3%), followed by the diagnostic area (4.3%).

The total social value that would be generated after the implementation of this set of proposals would amount to 271.94 million euros: 53.3% in the area of relapsing-remitting MS, 41.1% in the area of the progressive forms of MS, and 5.6% in the area of diagnosis.

**Fig. 3:**
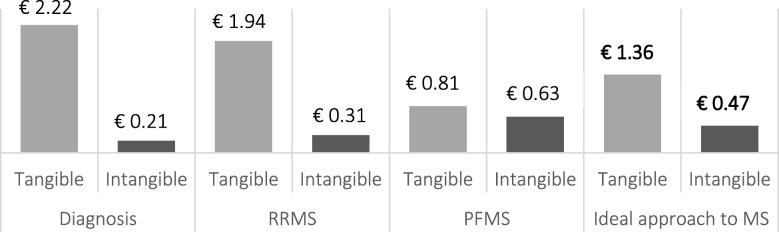
SROI ratio according to the areas of analysis and return typology. Abbreviations: MS, multiple sclerosis; RRMS, relapsing-relapsing MS; PFMS, progressive forms of MS, which include both primary progressive MS and secondary progressive MS; SROI, social return on investment

This implies that for every euro invested in the set of proposals included in the Project, 1.83 euros of social value would be generated. Of these, 74.2% would correspond to tangible returns, while 25.8% would be intangible and would include aspects such as the subjective experience of the patient to avoid an outbreak, the improvement of their emotional well-being or the burden of informal care. Figure 3. shows the social value that would be created by each area analysed while distinguishing the type of return.

The sensitivity analysis revealed that, under the assumptions considered, the potential social value would range from 1.59 euros to 2.15 euros for each euro invested (Table 4).

**Table 4:** Sensitivity analysis. Variation in the SROI ratio according to the scenario

Analysis area	Reference case	Worst scenario	Best scenario
Diagnosis	€ 2.43	€ 1.32	€ 3.54
Relapsing-remitting MS	€ 2.26	€ 2.14	€ 2.37
MS Progressive forms (primary progressive MS and secondary progressive MS)	€ 1.44	€ 1.25	€ 1.75
Total SROI	€ 1.83	€ 1.59	€ 2.15

Abbreviations: *SROI* social return on investment, *MS* multiple sclerosis

**Fig. 4:**
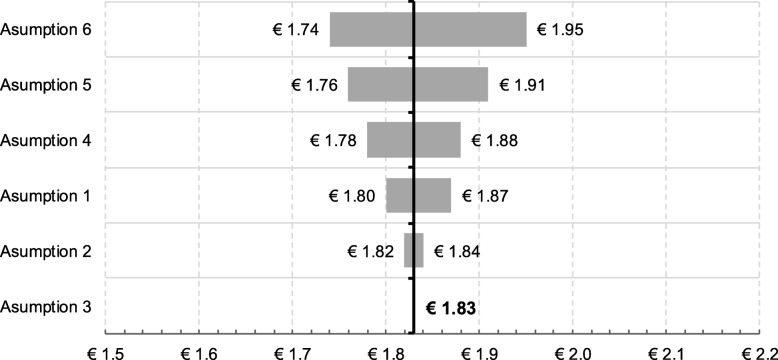
SROI ratio according to the variation of each variable included in the sensitivity analysis. Notes: Assumption 1. Percentage of disability reduction from moderate to mild in the incident patients, consequence of the reduction in the time to diagnosis; Assumption 2 Percentage of disability reduction from severe to moderate in the incident patients, consequence of the reduction in the time to diagnosis; Assumption 3 Percentage of cross-consultations avoided in neurology after direct consultation between Primary Care and Specialised Care professionals; Assumption 4. Percentage reduction of informal care hours, as a result of a better follow-up of patients and the slowing down of the progression of their illness; Assumption 5. Decrease in the percentage of patients who do not work because of MS; Assumption 6. Percentage of untreated SPMS patients, who could be treated

Figure 4 shows the ratio variation according to each variable included in the sensitivity analysis.

## Discussion

This study presents a set of proposals for improving the MS approach in the SNHS, as well as the evaluation of the potential social value that they would generate after its implementation.

Some of the proposals presented here had been previously collected, based on the chronic and complex nature of patients with MS and the specialised and continuous care they require [43, 44]. The first strength of the study would therefore be the confirmation of the needs already detected, based on the MWT consensus. However, in Spain, autonomous regions have autonomy in health management, so there may be different realities with regard to MS care [45]. Addressing these differences is a challenge to achieve equality in access to healthcare and ensure good health for the entire population and for patients with MS in particular. In this regard, the National Institute for Health and Care Excellence (NICE) in the United Kingdom proposes to evaluate health interventions from a dual approach of efficiency and equality that aims to ensure that all relevant benefits are taken into account (medical and non-medical and community), to help local authorities (and other organisations interested in improving the health of people) to better judge whether a public health intervention represents good value for money [46]. In addition, the inclusion of the perspective of patients within the MWT provides added value to the proposed set of measures and legitimises their implementation [47].

The results of the present study showed that a social value would be generated for patients with MS primarily, but also for their informal caregivers and the SNHS itself. In addition, said social value would be, in economic terms, almost twice the investment required for its implementation, with a ratio of 1:1.83 euros. However, we must keep in mind that this ratio is an abbreviated form of expressing all the potential social value. Hence, it is advisable to present it along with the rest of the information, mainly the theory of change [41].

Another strength of this study is the use of a mixed methodology, which includes both qualitative and quantitative analyses. The former refers to the theory of change, which explains and documents how an investment results in a certain impact. The latter is the process of quantitative analysis of investment and impact, which accounts for a broader concept of value that includes intangible aspects. In this phase, although it is impossible to maintain total objectivity, the provisions of the SROI guide were accurately and transparently followed [41].

The SROI method has hardly been used to evaluate different interventions in the area of public health, and never in relation to MS [37, 39, 40, 48, 49]. The challenge is not only to assess the current situation and create value around the management of patients with MS, but also to reflect on how decision making is performed in the SNHS and how the challenge of sustainability and efficiency is faced, since cutting back on health benefits represents a false economy [40].

This method poses the need to face the complexity of health care through knowledge. The traditional economic evaluation is based fundamentally on financial measurements that leave out a type of value that cannot be measured in this way. The SROI method focuses more on social value or impact than on expenditure, showing a broader type of value, and supports the collating of more comprehensive information on any intervention. The SROI method is not a substitute for other types of economic evaluation but potentially offers a more complete picture of outcomes which may support healthcare-related decision making.

This study is not without its limitations. First, there is no standardisation for the measurement of the social value inherent to health interventions. Second, in order to measure a broader concept of value, financial proxies were used to monetise that which does not have a market price. In this process, the subjective component is inevitable since two different experts could yield different results. Third, as it is a forecast study, its calculations, despite being referenced, are based on hypothetical scenarios. Therefore, the challenge remains to evaluate the real impact of these proposals once they are implemented and analyse the possible differences between both analyses. Fourth, having adopted a one-year timeframe may have biased the overview of the long-term impact of proposals. Since a forecast study implies unavoidable imprecision in data (estimated investment and return), having chosen a broader timeframe would have implied a higher imprecision. Finally, we assumed the entire population of MS patients would adhere to the set of proposals, but the whole adherence may be compromised due to proposals that demand time and effort from patients and caregivers (such as extra medical visits or tests). This might result in a lower SROI ratio which is hard to calculate as further studies about adherence to plans, in addition to treatments, are needed.

## Conclusions

The results of the present study show how patients with MS could improve their HRQoL while the SNHS could improve the efficiency of its health interventions. On the other hand, the proposals raised here could also generate impact outside the scope of MS and benefit, for example, patients with other illnesses, or health professionals. Although these impacts have not been quantified due to the magnitude of the study, the potential social value could be even greater.

## Supplementary information

**Additional file 1.** Patient survey.

## Data Availability

The datasets used and/or analysed during the current study, and data associated to the narrative review and patient survey are available from the corresponding author on reasonable request.

## Abbreviations

CSUR: Reference Centres, Services and Units
EQ-5D: EuroQol-5 Dimensions
HRQoL: Health-related quality of life
MRI: Magnetic resonance imaging
MS: Multiple sclerosis
MWT: Multidisciplinary Working Group
NICE: National Institute for Health and Care Excellence
PC: Primary care
PCM: Medicine / Primary care physician
PFMS: Progressive forms of MS
PPMS: Primarily progressive MS
RRMS: Relapsing-relapsing MS
SC: Specialised care
SNHS: Spanish National Health System
SPMS: Secondary Progressive MS
SROI: Social return on investment

## Footnotes

1 The drop-off applies only to SROI analysis with a timeframe of more than 1 year.

## References

[1] Ministerio de Sanidad, Servicios Sociales e Igualdad. Estrategia en Enfermedades Neurodegenerativas del Sistema Nacional de Salud. Madrid: Ministerio de Sanidad, Servicios Sociales e Igualdad; 2016. http://www.mscbs.gob.es/organizacion/sns/planCalidadSNS/pdf/Est_Neurodegenerativas_APROBADA_C_INTERTERRITORIAL.pdf. Accessed 24 Aug 2017.

[2] Déniz Cáceres A, Saavedra P, Marrero I (2011). Predicción del grado de minusvalía en pacientes con esclerosis múltiple. Rehabilitación.

[3] Servicio Canario de Salud. Guía de actuación en pacientes con esclerosis múltiple. In: Gobierno de Canarias; 2016. http://www3.gobiernodecanarias.org/sanidad/scs/contenidoGenerico.jsp?idDocument=aeab11ca-a4c8-11e6-acfb-b7af34d5e321&idCarpeta=a91550f4-75d0-11e2-bc0c-6512fc1bab5e. Accessed 24 Jan 2018.

[4] Fernández O, Luque G, San Román C, Bravo M, Dean G (1994). The prevalence of multiple sclerosis in the Sanitary District of Vélez-Málaga, southern Spain. Neurology.

[5] Fernández O, Fernández V, Guerrero M, León A, López-Madrona JC, Alonso A (2012). Multiple sclerosis prevalence in Malaga, southern Spain estimated by the capture-recapture method. Mult Scler J Lond.

[6] European Multiple Sclerosis Platform (2015). MS Barometer 2015. Raising the voice of people with MS.

[7] Ares B, Prieto JM, Lema M, Dapena D, Arias M, Noya M (2007). Prevalence of multiple sclerosis in Santiago de Compostela (Galicia, Spain). Mult Scler Clin Lab Res Lond.

[8] Carreón-Guarnizo E, Andreu-Reinón ME, Cerdán-Sánchez MC, Carrasco-Torres R, Hernández-Clares R, Prieto-Valiente L (2016). Prevalencia de la esclerosis múltiple en la Región de Murcia. Rev Neurol.

[9] Marrie RA, Cohen J, Stuve O, Trojano M, Sørensen PS, Reingold S (2015). A systematic review of the incidence and prevalence of comorbidity in multiple sclerosis: overview. Mult Scler Houndmills Basingstoke Engl.

[10] Marrie RA (2017). Comorbidity in multiple sclerosis: implications for patient care. Nat Rev Neurol.

[11] Marck CH, Neate SL, Taylor KL, Weiland TJ, Jelinek GA (2016). Prevalence of comorbidities, Overweight and Obesity in an International Sample of People with Multiple Sclerosis and Associations with Modifiable Lifestyle Factors. PLoS One.

[12] Marrie RA, Elliott L, Marriott J, Cossoy M, Blanchard J, Leung S (2015). Effect of comorbidity on mortality in multiple sclerosis. Neurology.

[13] Thormann A, Sørensen PS, Koch-Henriksen N, Laursen B, Magyari M (2017). Comorbidity in multiple sclerosis is associated with diagnostic delays and increased mortality. Neurology.

[14] Marrie RA, Horwitz R, Cutter G, Tyry T, Campagnolo D, Vollmer T (2009). Comorbidity delays diagnosis and increases disability at diagnosis in MS. Neurology..

[15] Sicras-Mainar A, Ruíz-Beato E, Navarro-Artieda R, Maurino J (2017). Comorbidity and metabolic syndrome in patients with multiple sclerosis from Asturias and Catalonia, Spain. BMC Neurol.

[16] Jones E, Pike J, Marshall T, Ye X (2016). Quantifying the relationship between increased disability and health care resource utilization, quality of life, work productivity, health care costs in patients with multiple sclerosis in the US. BMC Health Serv Res.

[17] Kobelt G, Thompson A, Berg J, Gannedahl M, Eriksson J, Group MS (2017). New insights into the burden and costs of multiple sclerosis in Europe. Mult Scler J.

[18] Oreja-Guevara C, Kobelt G, Berg J, Capsa D, Eriksson J, Platform EMS (2017). New insights into the burden and costs of multiple sclerosis in Europe: results for Spain. Mult Scler J.

[19] Meca-Lallana J, Mendibe M, Hernández-Clares R, Caminero AB, Mallada-Frechin J, Dávila-Gonzalez P (2016). Predictors of burden and depression among caregivers of relapsing-remitting MS patients in Spain: MS feeling study. Neurodegener Dis Manag.

[20] Labiano-Fontcuberta A, Mitchell AJ, Moreno-García S, Benito-León J (2015). Anxiety and depressive symptoms in caregivers of multiple sclerosis patients: the role of information processing speed impairment. J Neurol Sci.

[21] Oreja-Guevara C, Miralles A, García-Caballero J, Noval S, Gabaldón L, Esteban-Vasallo MD (2010). Diseño de una vía clínica para la atención a los pacientes con esclerosis múltiple. Neurología.

[22] Fernández O, Fernández V, Arbizu T, Izquierdo G, Bosca I, Arroyo R (2010). Characteristics of multiple sclerosis at onset and delay of diagnosis and treatment in Spain (the novo study). J Neurol.

[23] Rieckmann P, Centonze D, Elovaara I, Giovannoni G, Havrdová E, Kesselring J (2018). Unmet needs, burden of treatment, and patient engagement in multiple sclerosis: a combined perspective from the MS in the 21st century steering group. Mult Scler Relat Disord.

[24] Emerson J, Cabaj M (2000). Social return on investment. Mak Waves.

[25] Narrillos H (2012). Economía Social. Valoración y medición de la inversión social (método SROI).

[26] Tuan MT (2008). Measuring and/or estimating social value creation: insights into eight integrated cost approaches.

[27] Willis E, Semple AC, de Waal H (2018). Quantifying the benefits of peer support for people with dementia: a social return on investment (SROI) study. Dement Lond Engl.

[28] Jones C, Edwards RT, Windle G, Dementia, team I research, others. Social return on investment analysis of an art group for people with dementia Lancet 2014;384:S43.

[29] Merino M, Ivanova Y, Gómez-García T, Hidalgo-Vega Á, Díaz González F (2019). García de Vicuña R, et al. Proyecto SROI-AR Impacto clínico, asistencial, económico y social del abordaje ideal de la artritis reumatoide en comparación con el abordaje actual.

[30] González A, Ivanova Y, Jiménez M, Merino M, Hidalgo Á, Alfonso S (2016). Retorno Social de la Inversión de un abordaje ideal de la psoriasis.

[31] Durán Piñeiro G, Sánchez Carreira MC, Peña Gil C, Paredes-Galán E, Gómez Ruíz R, Lado Sestayo R (2015). El retorno económico y social de la e-interconsulta de cardiología en el área de Vigo. ICEDE Work Pap Ser ISSN 2254–7487.

[32] de Castro Carpeño J, Fírvida Pérez JL, Lianes Barragán P, Cobo Dols MÁ, Gil Gil JM, Carrato Mena A, et al. Cuantificando el beneficio de la sustitución por vinorelbina oral en los pacientes susceptibles de tratamiento con vinorelbina. Estudio del retorno social de la inversión. Rev Esp Econ Salud. 2018;13:336–53.

[33] Merino M, Jiménez M, Manito N, Casariego E, Ivanova Y, González-Domínguez A, et al. The social return on investment of a new approach to heart failure in the Spanish National Health System. ESC Heart Fail. 2020;n/a n/a. 10.1002/ehf2.12535. 10.1002/ehf2.12535 PMC7083495 31916416

[34] Lophongpanit P, Tongsiri S, Thongprasert N (2019). Social return on investment for patient treated by continuous ambulatory peritoneal Dialysis: a case study in Ubon Ratchathani Province, Thailand. Clin Outcomes Res.

[35] Jones Ray B, Ashurst Emily J, Atkey Jo, Duffy Barbara (2015). Older People Going Online: Its Value and Before-After Evaluation of Volunteer Support. Journal of Medical Internet Research.

[36] Scharlach AE (2015). Estimating the value of volunteer-assisted community-based aging services: a case example. Home Health Care Serv Q.

[37] Goudet S, Griffiths PL, Wainaina CW, Macharia TN, Wekesah FM, Wanjohi M (2018). Social value of a nutritional counselling and support program for breastfeeding in urban poor settings, Nairobi. BMC Public Health.

[38] Banke-Thomas Aduragbemi, Madaj Barbara, Kumar Shubha, Ameh Charles, van den Broek Nynke (2017). Assessing value-for-money in maternal and newborn health. BMJ Global Health.

[39] Banke-Thomas AO, Madaj B, Charles A, van den Broek N (2015). Social return on investment (SROI) methodology to account for value for money of public health interventions: a systematic review. BMC Public Health.

[40] Masters R, Anwar E, Collins B, Cookson R, Capewell S (2017). Return on investment of public health interventions: a systematic review. J Epidemiol Community Health.

[41] Nicholls J, Lawlor E, Neitzert E, Goodspeed T (2012). A guide to social return on investment.

[42] Instituto Nacional de Estadística. Índice de Precios de Consumo. Base 2016. Índices nacionales: general y de grupos ECOICOP. [Data file]. Retrieved from http://www.ine.es/jaxiT3/Tabla.htm?t=22553&L=0. Accessed 26 July 2017.

[43] Sociedad Española de Neurología (2016). Plan Estratégico Nacional para el Tratamiento Integral de las Enfermedades Neurológicas II (Pentien II).

[44] Berger Thomas, Adamczyk-Sowa Monika, Csépány Tünde, Fazekas Franz, Hojs Fabjan Tanja, Horáková Dana, Illes Zsolt, Klimová Eleonóra, Leutmezer Fritz, Rejdak Konrad, Rozsa Csilla, Šega Jazbec Saša, Szilasiová Jarmila, Turčáni Peter, Vachová Marta, Vécsei László, Havrdová Eva (2018). Management of multiple sclerosis patients in central European countries: current needs and potential solutions. Therapeutic Advances in Neurological Disorders.

[45] Urbanos-Garrido R (2016). La desigualdad en el acceso a las prestaciones sanitarias. Propuestas para lograr la equidad. Gac Sanit.

[46] National Institute for Health and Care Excellence (2013). How NICE measures value for money in relation to public health interventions.

[47] Rieckmann P, Boyko A, Centonze D, Elovaara I, Giovannoni G, Havrdová E (2015). Achieving patient engagement in multiple sclerosis: a perspective from the multiple sclerosis in the 21st century steering group. Mult Scler Relat Disord.

[48] Laing CM, Moules NJ (2017). Social return on investment: a new approach to understanding and advocating for value in healthcare. JONA J Nurs Adm.

[49] Dyakova M, Hamelmann C, Bellis MA, Besnier E, Grey CNB, Ashton K (2017). Investment for health and well-being: a review of the social return on investment from public health policies to support implementing the sustainable development goals by building on health 2020.

