# Supplementary material for: Social value of a set of proposals for the ideal approach of multiple sclerosis within the Spanish National Health System: a social return on investment study
Source: BMC Health Serv Res. 2020 Feb 4;20:84. doi: 10.1186/s12913-020-4946-8 (PMC7001370; doi:10.1186/s12913-020-4946-8)
Supplement: Supplementary file 1 — Additional file 1. Patient survey. [file 12913_2020_4946_MOESM1_ESM.pdf]

Additional file 1: Patient survey

PROJECT

I'EM

RecogEMos tu voz para avanzar en

ESCLEROSIS MÚLTIPLE*

SURVEY

SPONSORED BY

sefyp
SOCIEDAD ESPAÑOLA DE EDUCACIÓN PARA LA SALUD, FORMACIÓN Y DESARROLLO PROFESIONAL

*Translation: *We use your voice to advance in MULTIPLE SCLEROSIS*

RecogEMos tu voz para avanzar en Esclerosis Múltiple

Being active is the best way to cope with MS. Timely diagnosis and treatment help avoid or minimize the consequences of its progression and improve current results.

The project **I'EM, reality and needs of people with multiple sclerosis (MS) in Spain**, aims to analyse the socio-sanitary reality and the social impact of MS in our country, and to detect the basic needs of the current approach to MS. Furthermore, based on the outcomes, innovative proposals that contribute to the improvement of the quality of life of both patients and caregivers will be discussed.

Roche, together with EME, kindly ask for your collaboration by completing a survey that can be accessed at _______________. Please fill out the survey completely and in one single session, as this is the ideal way to do so. If, for any reason, this were impossible, you may also complete the survey in several stages as long as you complete each of the ten sections and use the save button located at the end of each section. Without a doubt, your collaboration is essential to make progress in Multiple Sclerosis.

Thank you very much for allowing us to capture your voice and, with it, continue to improve MS care.

ACCESS DATA

1. Sex
   - ○ Female
   - ○ Male

2. ¿What type of multiple sclerosis you are currently suffering from?
   - ○ Primary-Progressive ME (continuous or gradual progression of the disease, there may or may not be outbreaks)
   - ○ Relapsing-Remitting MS (unpredictable outbreaks with full or partial recovery)
   - ○ Secondary-Progressive MS (starts with Relapsing-Remitting, followed by progression with or without outbreaks)
   - ○ I don't know
   - ○ I don't have multiple sclerosis **(END OF QUESTIONNAIRE)**

3. How long you have been diagnosed with MS?
   - ○ Less than 1 year
   - ○ Between 1 and 2 years
   - ○ Between 3 and 4 years
   - ○ Between 5 and 9 years
   - ○ 10 or more years

GENERAL QUESTIONS

4. Age: ☐☐

5. Education
   - ○ No education
   - ○ Primary studies
   - ○ Secondary studies
   - ○ Higher education

6. Marital status
   - ○ Single
   - ○ Married or domestic partnership
   - ○ Separated or divorced
   - ○ Widowed

7. Current employment situation
   - ○ Employed by others
   - ○ Self-employment
   - ○ On a temporary work leave
   - ○ Pre-retirement
   - ○ Retired
   - ○ Unemployed
   - ○ Permanent disability (dependency)
   - ○ Housework
   - ○ Another situation (specify): ________________

8. Habitat of habitual residence
   - Small Rural (2,000 inhabitants or less)
   - Intermediate Rural (2,001-10,000 inhabitants)
   - Large Rural (10,001-40,000 inhabitants)
   - Urban (more than 40,000 inhabitants)

FAMILY

9. Who do you usually live with?
   (MULTIPLE CHOICE QUESTION, UNLESS YOU RESPOND 'ALONE')
   - Alone
   - Parents
   - Children
   - Brothers and/or sisters
   - Spouse/partner
   - Other relatives
   - Non-family members

10. ¿Where do you usually live?
    - In a house/flat
    - In a residence
    - In another type of care centre

11. If you live in a house/flat, ¿are you the owner (ore one of the owners)?
    - Yes
    - No
    - Does not apply (I live in a residence or another type of care centre)

NEED FOR INFORMAL OR PREFESSIONAL CARE

12. What kind of attention and care you getting from the people who live with you?
    (MULTIPLE CHOICE QUESTION, UNLESS YOU RESPOND TO ANY OF THE FIRST TWO OPTIONS)
    - None, I am independent
    - Does not apply (I live in a residence or residential centre)
    - Attention to daily tasks
    - Accompaniment to medical visits
    - Treatment follow-up (for example: remind intake schedule, facilitate intakes, inform of adverse effects, interactions, etc.)
    - Information and education regarding coping with the disease
    - Others (specify): ___________________________________

13. What kind of attention and care are you getting from those family members that don't live with you?
    - Does not apply
    - Attention to daily tasks
    - Accompaniment to medical visits
    - Treatment follow-up (for example: remind intake schedule, facilitate intakes, inform of adverse effects, interactions, etc.)
    - Information and education regarding coping with the disease
    - Others (specify): ___________________________________

14. Regardless of living with or without your family, do you need the help of an outside caregiver?
   - ○ Yes
   - ○ No

14.1 If so, what kind of attention and care is this professional caregiver providing?
   - ○ Attention to daily tasks
   - ○ Accompaniment to medical visits
   - ○ Treatment follow-up (for example: remind intake schedule, facilitate intakes, inform of adverse effects, interactions, etc.)
   - ○ Information and education regarding coping with the disease
   - ○ Others (specify): ___________________________________

PHYSICAL AND PSYCHOLOGICAL FACTORS

15. How do you perceive your current health status after being diagnosed (rate from 1 to 10, with 1 being the least worsening and 10 being the most worsening)?

Not worse									A lot worse
1	2	3	4	5	6	7	8	9	10
☐	☐	☐	☐	☐	☐	☐	☐	☐	☐

16. How do you perceive your current health status compared to twelve months ago (rate from 1 to 10, with 1 being the least worsening and 10 being the most worsening)?

Not worse									A lot worse
1	2	3	4	5	6	7	8	9	10
☐	☐	☐	☐	☐	☐	☐	☐	☐	☐

17. Regardless of your previous health status, how would you rate your current general quality of life (rate from 1 to 10, with 1 being the lowest quality of life and 10 being the highest quality of life)?

Lowest Quality of life									Highest Quality of life
1	2	3	4	5	6	7	8	9	10
☐	☐	☐	☐	☐	☐	☐	☐	☐	☐

18. Which of the following do you think has been most affected by the disease since diagnosis?
   - ○ Mobility
   - ○ Activities of daily living
   - ○ Hygiene and personal care
   - ○ Others (specify): _____________________________________

19. How do you consider the following aspects to affect your current quality of life (QoL) (rank from 1 to 10, with 1 being the value that least affects the QoL and 10 the value that most affects the QoL)?

	Aspect which LEAST AFFECTS my quality of life 1	2	3	4	5	6	7	8	9	Aspect which MOST AFFECTS my quality of life 10
Pain/discomfort	☐	☐	☐	☐	☐	☐	☐	☐	☐	☐
Anxiety/depression	☐	☐	☐	☐	☐	☐	☐	☐	☐	☐
Feeling of fatigue (loss of strength and skill)	☐	☐	☐	☐	☐	☐	☐	☐	☐	☐
Difficulty with rest and night's sleep	☐	☐	☐	☐	☐	☐	☐	☐	☐	☐
Sensory problems (extremity numbness, tingling…)	☐	☐	☐	☐	☐	☐	☐	☐	☐	☐
Loss of cognitive function (attention, reasoning, memory…)	☐	☐	☐	☐	☐	☐	☐	☐	☐	☐
Loss of balance (feeling of instability)	☐	☐	☐	☐	☐	☐	☐	☐	☐	☐
Sexual function	☐	☐	☐	☐	☐	☐	☐	☐	☐	☐
Urination problems	☐	☐	☐	☐	☐	☐	☐	☐	☐	☐
Visual impairment	☐	☐	☐	☐	☐	☐	☐	☐	☐	☐

FIRST SYMPTOMS AND DIAGNOSIS

20. Do you remember how much time passed between the first symptoms and the diagnosis?
    - ○ Less than 1 month
    - ○ From 1 to 5 months
    - ○ From 6 to 11 months
    - ○ From 1 to 3 years
    - ○ More than 3 years

21. Among the following disease warning signs that may have appeared, could you rate their importance to you (rate from 1 to 10, with 1 being of minor importance and 10 being of major importance)?

	Minor Importance<br>1	2	3	4	5	6	7	8	9	Major Importance<br>10
Feeling of fatigue (loss of strength and skill)	☐	☐	☐	☐	☐	☐	☐	☐	☐	☐
Sensory decrease (extremity numbness, tingling…)	☐	☐	☐	☐	☐	☐	☐	☐	☐	☐
Loss of cognitive function (attention, reasoning, memory…)	☐	☐	☐	☐	☐	☐	☐	☐	☐	☐
Loss of balance (feeling of instability)	☐	☐	☐	☐	☐	☐	☐	☐	☐	☐
Visual impairments (blurry or double vision)	☐	☐	☐	☐	☐	☐	☐	☐	☐	☐
Pain/discomfort	☐	☐	☐	☐	☐	☐	☐	☐	☐	☐

22. Who did you first turn to at the first signs?
   - ○ General practitioner
   - ○ Neurologist
   - ○ Another specialist (specify): ___________________

23. Who diagnosed the disease?
   - ○ General practitioner
   - ○ Neurologist
   - ○ Another specialist (specify): ___________________

24. What tests were performed to establish the diagnosis of MS? (MULTIPLE CHOICE POSSIBILITY)
   - ○ Medical report
   - ○ Physical exploration
   - ○ Blood test
   - ○ Lumbar Puncture
   - ○ Brain Magnetic Resonance Imaging (MRI) test
   - ○ Evoked Potentials
   - ○ Others (specify): ___________________

25. How long did it take between the first visit to your doctor and the first MRI test?
   - ○ Does not apply, no MRI test was performed
   - ○ Less than 1 month
   - ○ From 1 to 4 months
   - ○ From 5 to 8 months
   - ○ From 9 to 12 months
   - ○ More than 1 year

26. How would you rate the information received regarding the MRI test as a diagnostic tool?
   - ○ Very good
   - ○ Good
   - ○ Regular
   - ○ Bad
   - ○ Very bad

27. Regardless of whether or not you had an MRI test for the initial diagnosis of the disease, has this test been used again to assess the progression of the disease?
   - ○ Yes
   - ○ No

27.1 If so, how many times has this test been performed?

1	2	3	4	5	6	More than 6
☐	☐	☐	☐	☐	☐	☐

27.2 Approximately, how often is the MRI test performed to assess the progress of the disease?
   - ○ 6 months
   - ○ 1 year
   - ○ 2 years
   - ○ 3 years
   - ○ More than 3 years

28. How would you rate the information you currently have about the MRI test in order to evaluate the follow-up of the disease?
   - ○ Very good
   - ○ Good
   - ○ Regular
   - ○ Bad
   - ○ Very bad

29. How would you rate the inconveniences (timetables, times, noise, access) when performing the test (rate from 1 to 10, with 1 being the least difficult and 10 the most difficult)?

Least Difficult 1	2	3	4	5	6	7	8	9	Most Difficult 10
☐	☐	☐	☐	☐	☐	☐	☐	☐	☐

INFORMATION ON THE DISEASE

30. What knowledge did you have about MS before diagnosis?
   - ○ Very good
   - ○ Good
   - ○ Regular
   - ○ Bad
   - ○ Very bad
   - ○ No knowledge

31. How would you rate your current knowledge on the disease?
    - ○ Very good
    - ○ Good
    - ○ Regular
    - ○ Bad
    - ○ Very bad

32. What is your main source of information on the disease?
    - ○ General practitioner
    - ○ Neurologist
    - ○ Nurse
    - ○ Patient associations and channels (web, newsletter, etc.)
    - ○ Friends/family
    - ○ Caregivers
    - ○ Internet
    - ○ Others (specify): ____________

33. Which would you consider to be more reliable?
    - ○ General practitioner
    - ○ Specialist
    - ○ Nurse
    - ○ Patient associations
    - ○ Friends/family
    - ○ Caregivers
    - ○ Internet
    - ○ Others (specify): ____________

34. To what extent do you consider the symptoms to be the most decisive element for the progress of the disease (rate from 1 to 10, with 1 being the least decisive and 10 the most)?

Least Decisive									Most Decisive
1	2	3	4	5	6	7	8	9	10
☐	☐	☐	☐	☐	☐	☐	☐	☐	☐

35. To what extent do you agree with the following sentence?

"AS A RESULT OF DISEASE PROGRESSION, THERE MAY BE LOSS OF BRAIN TISSUE EVEN IN THE ABSENCE OF DISEASE SYMPTOMS"
    - ○ Strongly agree
    - ○ Agree
    - ○ Don't agree much
    - ○ Don't agree at all

36. To what extent do you agree with the following sentence?

"EARLY DIAGNOSIS AND RAPID TREATMENT INITIATION CAN MINIMIZE THE PROGRESSION OF THE DISEASE"
    - ○ Strongly agree
    - ○ Agree
    - ○ Don't agree much
    - ○ Don't agree at all

TREATMENT

37. What type(s) of health care are you currently receiving?
(IF APPLICABLE, YOU MAY CHECK MORE THAN ONE OPTION)
- ○ Public
- ○ Private
- ○ Combined
- ○ Other (specify): ____________________

38. Are you currently on MS course-modifying therapy?
- ○ Yes
- ○ No

38.1 If so, how much time has elapsed between the diagnosis and the start of the treatment?
- ○ It was immediate (on the same day)
- ○ Less than 1 month
- ○ From 1 to 6 months
- ○ From 7 to 12 months
- ○ More than 1 year

39. Since the diagnosis and the first treatment, have you undergone any changes in treatment?
- ○ Yes
- ○ No

39.1 If so, how many treatment changes have you had since the first?

1	2	3	4	5	6	More than 6
☐	☐	☐	☐	☐	☐	☐

39.2 What was the main reason for the change (if you had more than one change, please refer to the last change)?
(POSSIBILITY OF MARKING MORE THAN ONE OPTION)
- ○ Lack of effectiveness
- ○ Toxicity/adverse effects
- ○ Inconvenience of the route of administration
- ○ Others (specify): ______________________

39.3 Who decided to change the treatment (if you had more than one change, please refer to the last change)?
- ○ My doctor
- ○ Me
- ○ It was a joint decision between me and my doctor
- ○ Others (specify): ______________________

40. How would you rate your level of satisfaction with your current treatment (rate from 1 to 10, with 1 being the lowest level of satisfaction and 10 the highest level of satisfaction)?

Lowest Satisfaction									Highest Satisfaction
1	2	3	4	5	6	7	8	9	10
☐	☐	☐	☐	☐	☐	☐	☐	☐	☐

41. What is your preferred administration route for the current treatment?

Parenteral (intravenous)	Parenteral (intramuscular)	Subcutaneous	Oral
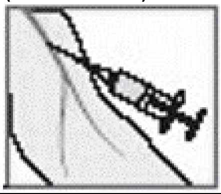	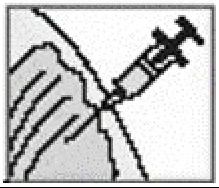	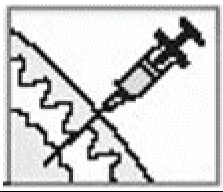	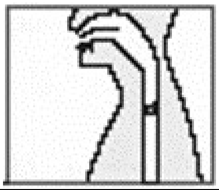

42. How would you rate your knowledge on the treatments you have received or are currently taking?
    - ○ Very good
    - ○ Good
    - ○ Regular
    - ○ Bad
    - ○ Very bad

43. And on the possibilities of new treatments in the near future?
    - ○ Very good
    - ○ Good
    - ○ Regular
    - ○ Bad
    - ○ Very bad

44. To what extent do you feel you have the ability to be involved in treatment decisions and share responsibility for treatment choice with the physician (degree of empowerment)?
    - ○ Very much
    - ○ Somewhat
    - ○ Fair
    - ○ Not much
    - ○ Not at all

45. At the present time, to what extent does your doctor take into account your therapeutic preferences?
    - ○ Very much
    - ○ Somewhat
    - ○ Fair
    - ○ Not much
    - ○ Not at all

46. Which of the following benefits do you most value?
    - ○ Effectiveness (deceleration of disease progression)
    - ○ Safety (minimizing toxicity and organic damage/adverse effects)
    - ○ Comfort of administration route
    - ○ Others (specify): _________________

47. To what extent do you agree with the following sentence?

"TREATMENT CAN SLOW THE PROGRESSION OF THE DISEASE REGARDLESS OF SYMPTOMS"
    - ○ Strongly agree
    - ○ Agree
    - ○ Don’t agree much
    - ○ Don’t agree at all

48. To what extent do you agree with the following sentence?

"REGULAR MONITORING OF DISEASE ACTIVITY BY MRI CAN GIVE US AN EARLY WARNING THAT MS IS NOT RESPONDING WELL TO TREATMENT."

- ○ Strongly agree
- ○ Agree
- ○ Don't agree much
- ○ Don't agree at all

49. Have you ever made the decision to stop treatment permanently regardless of your doctor's decision?

- ○ Yes
- ○ No

RELATIONSHIP WITH OTHER HEALTH PROFESSIONALS

50. Given your experience, how would you rate the health care received from the primary care physician (general practitioner) (rate from 1 to 10, with 1 being the lowest value and 10 the highest value)?

DOES NOT APPLY	Lowest Value									Highest Value
☐	1	2	3	4	5	6	7	8	9	10
	☐	☐	☐	☐	☐	☐	☐	☐	☐	☐

51. Given your experience, how would you rate the health care received from the neurologist (rate from 1 to 10, with 1 being the lowest value and 10 the highest value)?

DOES NOT APPLY	Lowest Value									Highest Value
☐	1	2	3	4	5	6	7	8	9	10
	☐	☐	☐	☐	☐	☐	☐	☐	☐	☐

52. Given your experience, how would you rate the health care received from the MRI expert (rate from 1 to 10, with 1 being the lowest value and 10 the highest value)?

DOES NOT APPLY	Lowest Value									Highest Value
☐	1	2	3	4	5	6	7	8	9	10
	☐	☐	☐	☐	☐	☐	☐	☐	☐	☐

53. Given your experience, how would you rate the health care received from the nursing personnel (rate from 1 to 10, with 1 being the lowest value and 10 the highest value)?

DOES NOT APPLY	Lowest Value									Highest Value
☐	1	2	3	4	5	6	7	8	9	10
	☐	☐	☐	☐	☐	☐	☐	☐	☐	☐

54. Which of the different healthcare professionals has the greatest influence on your behaviour regarding the disease and its treatment?

- ○ General practitioner
- ○ Neurologist
- ○ Nursing personnel
- ○ Another healthcare/social professional (specify): ___________________________

55. What kind of relationship do you have with him/her?
   - ○ Paternalistic (the healthcare professional decides and gives recommendations to be followed by the patient)
   - ○ Co-responsibility (decisions are made jointly)

56. In either case, how satisfied are you with this type of relationship (rate from 1 to 10, with 1 being the lowest level of satisfaction and 10 the highest)?

Lowest Satisfaction									Highest Satisfaction
1	2	3	4	5	6	7	8	9	10
☐	☐	☐	☐	☐	☐	☐	☐	☐	☐

HEALTHCARE SYSTEM

57. In your opinion, how high is the priority of the National Health System for MS (rate from 1 to 10, with 1 being the lowest priority and 10 the highest)?

Lowest Priority									Highest Priority
1	2	3	4	5	6	7	8	9	10
☐	☐	☐	☐	☐	☐	☐	☐	☐	☐

58. To what extent do you consider that MS patients in Spain have the ability to influence health authorities regarding the availability of diagnostic tests funded by the National Health System?
   - ○ Very much
   - ○ Somewhat
   - ○ Fair
   - ○ Not much
   - ○ Not at all

59. To what extent do you consider that MS patients in Spain have the ability to influence health authorities regarding the availability of treatments funded by the National Health System?
   - ○ Very much
   - ○ Somewhat
   - ○ Fair
   - ○ Not much
   - ○ Not at all

60. To what extent do you consider that MS patients in Spain have the ability to influence health authorities regarding the availability of rehabilitative measures funded by the National Health System?
   - ○ Very much
   - ○ Somewhat
   - ○ Fair
   - ○ Not much
   - ○ Not at all

61. To what extent do you think the National Health System is prepared and has sufficient capacity to care for patients with MS?
   - ○ Very much
   - ○ Somewhat
   - ○ Fair
   - ○ Not much
   - ○ Not at all

62. To what extent would you like to have the following free healthcare services from the NHS (mark a number from 1 to 10, taking into account that 1 represents the lowest degree of satisfaction and 10 the highest degree of satisfaction)?

	Lowest Satisfaction 1	2	3	4	5	6	7	8	9	Highest Satisfaction 10
Physiotherapy programs (in hospital or at home)	☐	☐	☐	☐	☐	☐	☐	☐	☐	☐
Psychotherapy programs (availability of MS-oriented/specialized psychologist who can further provide support at home)	☐	☐	☐	☐	☐	☐	☐	☐	☐	☐
Physiotherapy, including pelvic floor exercises, resistance, balance, etc.	☐	☐	☐	☐	☐	☐	☐	☐	☐	☐
External caregivers, especially for the most dependent patients	☐	☐	☐	☐	☐	☐	☐	☐	☐	☐
Hotline allowing direct and fast contact with a specialist	☐	☐	☐	☐	☐	☐	☐	☐	☐	☐
Improved access to healthcare institutions (ramps, parking areas, etc.)	☐	☐	☐	☐	☐	☐	☐	☐	☐	☐
Availability of specific MS units in healthcare centres	☐	☐	☐	☐	☐	☐	☐	☐	☐	☐

63. How do you consider the information received to maintain a healthy lifestyle?
   - ○ Very good
   - ○ Good
   - ○ Regular
   - ○ Bad
   - ○ Very bad

64. How do you consider the information received regarding rights and services associated with the situation of disability and the impact of the disease on the life of the patient?
   - ○ Very good
   - ○ Good
   - ○ Regular
   - ○ Bad
   - ○ Very bad

SIGNS AND SYMPTOMS AFFECTED

65. The following questions are in relation to whether or not MS has generated different signs and symptoms. If so, you are asked what is the maximum you would be willing to pay their improvement.

**Has MS caused you any problems with…**	**Yes / No**	**If Yes**, what would be your maximum **willingness to pay** for each problem related to MS?
65.1 … your sensory state (tingling, swelling, limb numbness…)?	☐Yes<br>☐No	___________ Euros<br>☐I don't know<br>☐I prefer not to answer
65.2 … your motor state (spasticity, tremors, walking, other mobility problems…)?	☐Yes<br>☐No	___________ Euros<br>☐ I don't know<br>☐ I prefer not to answer
65.3 … your balance and coordination?	☐Yes<br>☐No	___________ Euros<br>☐ I don't know<br>☐ I prefer not to answer
65.4… feeling fatigue and y lack of energy?	☐Yes<br>☐No	___________ Euros<br>☐ I don't know<br>☐ I prefer not to answer
65.5… your vision?	☐Yes<br>☐No	___________ Euros<br>☐I don't know<br>☐ I prefer not to answer
65.6… your ability to speak?	☐Yes<br>☐No	___________ Euros<br>☐I don't know<br>☐ I prefer not to answer
65.7… urinary incontinence?	☐Yes<br>☐No	___________ Euros<br>☐I don't know<br>☐ I prefer not to answer
65.8… sphincter control?	☐Yes<br>☐No	___________ Euros<br>☐I don't know<br>☐ I prefer not to answer
65.9… your cognitive state (concentration, memorization…)?	☐Yes<br>☐No	___________ Euros<br>☐I don't know<br>☐ I prefer not to answer
65.10… emotional state (depression, anxiety…)?	☐Yes<br>☐No	___________ Euros<br>☐I don't know<br>☐ I prefer not to answer
65.11… other symptoms (pain, discomfort, weakness…)?	☐Yes<br>☐No	___________ Euros<br>☐I don't know<br>☐ I prefer not to answer

66. The following questions are in relation to whether or not MS has generated problems in different areas of living. If so, you are asked what is the maximum you would be willing to pay their improvement.

**Has MS caused you any problems with…**	**Yes / No**	**If Yes**, what would be your maximum **willingness to pay** for each problem related to MS?
66.1 … your self-care?	☐Yes<br>☐No	___________ Euros<br>☐I don't know<br>☐I prefer not to answer
66.2… your romantic relationships?	☐Yes<br>☐No<br>☐Not apply *(I am single)*	_______________ Euros<br>☐ I don't know<br>☐ I prefer not to answer
66.3… your relationship with your family?	☐Yes<br>☐No	___________ Euros<br>☐ I don't know<br>☐ I prefer not to answer
66.4… your social relationships?	☐Yes<br>☐No	___________ Euros<br>☐ I don't know<br>☐ I prefer not to answer
66.5…your leisure environment?	☐Yes<br>☐No	___________ Euros<br>☐ I don't know<br>☐ I prefer not to answer
66.6… work environment?	☐Yes<br>☐No<br>☐Not apply	_______________ Euros<br>☐ I don't know<br>☐ I prefer not to answer

67. What is the maximum you would be willing to pay for…?

67.1 … preventing an outbreak?	___________ Euros<br>☐ I don't know<br>☐ I prefer not to answer
67.2 … having been diagnosed earlier (preventing a delay in diagnosis)?	___________ Euros<br>☐ I don't know<br>☐ I prefer not to answer

DISEASE RECOGNITION

68. What degree of disability have you been officially recognized with? (from 0% to 100%): __ __ __ %

69. What financial compensation do you receive from the State mainly because of your MS?
    - ○ I am not receiving any financial compensation from the State
    - ○ 500 euros or less
    - ○ Between 501 and 1,000 Euros
    - ○ Between 1,001 and 1,500 Euros
    - ○ Between 1,501 and 2,000 Euros
    - ○ More than 2,000 Euros
    - ○ I don't know
    - ○ I prefer not to answer
